# Myostatin-2 gene structure and polymorphism of the promoter and first intron in the marine fish *Sparus aurata*: evidence for DNA duplications and/or translocations

**DOI:** 10.1186/1471-2156-12-22

**Published:** 2011-02-01

**Authors:** Elisabeth Nadjar-Boger, Bruria Funkenstein

**Affiliations:** 1 National Institute of Oceanography, Israel Oceanographic and Limnological Research, Tel-Shikmona, P.O.B 8030, Haifa 31080, Israel

## Abstract

**Background:**

Myostatin (MSTN) is a member of the transforming growth factor-ß superfamily that functions as a negative regulator of skeletal muscle development and growth in mammals. Fish express at least two genes for *MSTN*: *MSTN-1 *and *MSTN-2*. To date, *MSTN-2 *promoters have been cloned only from salmonids and zebrafish.

**Results:**

Here we described the cloning and sequence analysis of *MSTN-2 *gene and its 5' flanking region in the marine fish *Sparus aurata *(sa*MSTN-2*). We demonstrate the existence of three alleles of the promoter and three alleles of the first intron. Sequence comparison of the promoter region in the three alleles revealed that although the sequences of the first 1050 bp upstream of the translation start site are almost identical in the three alleles, a substantial sequence divergence is seen further upstream. Careful sequence analysis of the region upstream of the first 1050 bp in the three alleles identified several elements that appear to be repeated in some or all sequences, at different positions. This suggests that the promoter region of sa*MSTN-2 *has been subjected to various chromosomal rearrangements during the course of evolution, reflecting either insertion or deletion events. Screening of several genomic DNA collections indicated differences in allele frequency, with allele 'b' being the most abundant, followed by allele 'c', whereas allele 'a' is relatively rare. Sequence analysis of sa*MSTN-2 *gene also revealed polymorphism in the first intron, identifying three alleles. The length difference in alleles '1R' and '2R' of the first intron is due to the presence of one or two copies of a repeated block of approximately 150 bp, located at the 5' end of the first intron. The third allele, '4R', has an additional insertion of 323 bp located 116 bp upstream of the 3' end of the first intron. Analysis of several DNA collections showed that the '2R' allele is the most common, followed by the '4R' allele, whereas the '1R' allele is relatively rare. Progeny analysis of a full-sib family showed a Mendelian mode of inheritance of the two genetic loci. No clear association was found between the two genetic markers and growth rate.

**Conclusion:**

These results show for the first time a substantial degree of polymorphism in both the promoter and first intron of *MSTN-2 *gene in a perciform fish species which points to chromosomal rearrangements that took place during evolution.

## Background

Myostatin (MSTN) is a member of the transforming growth factor (TGF)-β superfamily and a negative regulator of skeletal muscle growth in mammals [reviewed in [1]]. The inhibition of muscle growth is believed to occur by negative regulation of both myoblast proliferation and differentiation [2-7]. MSTN knockout mice have two- to threefold greater muscle mass than their wild type littermates, mainly due to an increase in the number of muscle fibers (hyperplasia) and the diameter of the fibers (hypertrophy) [8]. Transgenic mice carrying a dominant negative *MSTN *showed up to a 35% increase in skeletal muscle mass and this increase was the result of muscle hypertrophy [9]. The phenomenon known as "double muscling" of increased muscle mass found in several cattle breeds is associated with natural mutations in the *MSTN *coding sequence [10-13]. Muscle mass gain as a result of a mutation in the *MSTN *gene was reported recently also in dogs [14], sheep [15,16] and a child [17].

The key role of MSTN in muscle growth and its potential application in animal husbandry encouraged the cloning of *MSTN *cDNAs and genes from numerous fish species of value to aquaculture [[18] and references therein; [19] and references therein]. These studies have shown that in contrast to mammals and chicken, fish possess at least two distinct *MSTN *genes with differential expression [reviewed in [20]], probably as a result of gene duplication. A comprehensive phylogenetic analysis [21] suggested that several of the salmonid genes, formerly identified as *MSTN-2*, are actually *MSTN-1 *orthologs, and were re-classified as *MSTN-1a *and *MSTN-1b*. However, sequence similarity and clustering supported the existence of *MSTN-2 *in the gilthead sea bream (*Sparus aurata*), shi drum (*Umbrina cirrosa*), Fugu (*Takifugu rubripes*) and zebrafish (*Danio rerio*). Subsequent studies identified 'true' *MSTN-2 *genes also in the rainbow trout [22]. These studies have also shown that, in contrast to mammals, fish express MSTN not only in red and white muscle, but also in other tissues, suggesting that MSTN might function in fish not only in growth but also in other physiological processes. The pattern of expression of the two genes differs between various fish species. In *S. aurata MSTN-1 *is ubiquitously expressed in various tissues including muscle, whereas *MSTN-2 *mRNA is expressed mainly in the brain including olfactory and optic lobes [23,24]. By contrast, in other fish species (coho salmon (*Oncorhynchus kisutch*) [25], Atlantic salmon (*Salmo salar*) [26], zebrafish [27] and rainbow trout (*Oncorhynchus mykiss*) [22]) *MSTN-2 *is expressed not only in brain but also in other tissues, including muscle.

The goal of our research is to develop new technology based on MSTN in order to enhance fish muscle growth. To gain insight into the regulation of *MSTN *genes in fish, we recently reported the cloning and characterization of *MSTN-1 *promoter from a commercially very important marine fish species in the Mediterranean region, *S. aurata*. In that study, we showed that the proximal promoter is highly conserved in fish. We also provided experimental evidence for the function of the 5' flanking region of *MSTN-1 *gene as promoter using luciferase reporter gene assay and A204 cells [28]. The current study was undertaken in order to expand our knowledge of MSTN-2 in fish by characterizing the structure of the *MSTN-2 *gene and its 5' flanking region in *S. aurata*. We describe the genomic organization of the *MSTN-2 *gene and promoter in *S. aurata *and the discovery of polymorphism of both the promoter and the first intron, probably as a result of chromosomal re-arrangements. These studies also show that the various alleles are inherited in a Mendelian manner.

## Methods

### Fish and tissues

*S. aurata *fish for this study were obtained from several sources as detailed below. In addition, three full-sib families of *S. aurata *were produced at The National Center of Mariculture, Eilat, Israel by Drs. S. Gorshkov and G. Gorshkova using gametes from stripped fish and artificial fertilization. Cross 1 (sire PIT tag #0661; dam #5E4D) was chosen since the parents were found to be heterozygotes for the two loci examined (Table 1). A progeny, consisting of 27 fingerlings (one group of small fingerlings with mean body weight 0.16 gr and a second group of large fingerlings with mean body weight of 1 gr) was used for analysis of Mendelian inheritance.

**Table 1:** Segregation of sa*MSTN-2 *alleles of the promoter and intron 1 in progeny of a full-sib family (cross 1)

PROMOTER					INTRON1				
	**PARENTS**								
		
	Dam (#5E4D)		Sire (#0661)		Dam (#5E4D)			Sire (#0661)	
		
	a/b		b/c		1R/2R			2R/4R	

**PROGENY**									

		Expected Number (frequency)	Observed Number (frequency)	Contribution to χ^2^			Expected Number (frequency)	Observed Number (frequency)	Contribution to χ^2^

ALLELES	a	13 (25%)	11 (21%)	0.308	ALLELES	1R	13.5 (25%)	12 (22%)	0.166
			
	b	26 (50%)	31 (60%)	0.962		2R	27 (50%)	32 (59%)	0.926
			
	c	13 (25%)	10 (19%)	0.692		4R	13.5 (25%)	10 (19%)	0.907

	TOTAL	52 (100%)	52 (100%)	**1.962** **not** **significant**		TOTAL	54 (100%)	54 (100%)	**1.999** **not** **significant**

GENOTYPES	a/b	6.5 (25%)	3 (11%)	1.885	GENOTYPES	1R/2R	6.75 (25%)	4 (15%)	1.120
			
	b/b	6.5 (25%)	13 (50%)	6.500		2R/2R	6.75 (25%)	13 (48%)	5.787
			
	a/c	6.5 (25%)	8 (31%)	0.346		1R/4R	6.75 (25%)	8 (30%)	0.231
			
	b/c	6.5 (25%)	2 (8%)	3.115		2R/4R	6.75 (25%)	2 (7%)	3.343

	TOTAL	26 (100%)	26 (100%)	**11.846** **significant**		TOTAL	27 (100%)	27 (100%)	**10.481** **significant**

The results show no statistically significant deviation from Mendelian inheritance for alleles (0.9 >> P >> 0.10), but statistically significant deviation from Mendelian inheritance for genotypes (P << 0.05)

Five collections of DNA samples from *S. aurata *were used for the analysis of *MSTN-2 *gene and promoter polymorphism: fingerlings from two hatcheries in Israel: Ardag Red Sea Mariculture, Eilat (60 individuals, 3-month-old fingerlings spawned at the same time), and The Israel Salt Company, Atlit (52 individuals). The third collection, from Instituto Nacional de Investigaçao das Pescas (INIP; Olhão, Portugal), consisted of 19 individuals; the fourth collection consisted of 23 individuals from a wild population caught near Ancona, Italy. The fifth collection ('GC' collection) consisted of 15 individuals of unknown geographic origin, from Mevo'ot Yam School, Michmoret, Israel. The fingerlings from the two Israeli hatcheries belonged to two size groups, in order to test for a possible association between growth and allele/genotype distribution. The Atlit hatchery had a recently established population (3 generations) of *S. aurata *from different geographical origins; the collection consisted of 25 fingerlings with a "Normal" phenotype (0.1 g body weight), and 27 individuals with the largest size "Jumper" phenotype [29] (1 g body weight). The second collection, from the Ardag hatchery, was from a partially domesticated population (5-10 generations in captivity) that underwent three generations of growth selection and consisted of 30 fingerlings with the largest size "Large" phenotype (4.3 g) and 30 individuals with the smallest size "Small" phenotype (0.25 g). The fish making up the size groups were identified during body weight selection by the fish farmers. This practice is routinely done at the fish farms to overcome behavioral problems (cannibalism, competition for food, etc.) which result from differences in body size. Therefore, similar size groups were kept in separate tanks until sampling.

Fin clips were snap-frozen in liquid nitrogen and kept at -70°C until DNA was extracted. In few experiments, fin clips were put in absolute ethanol and kept at 4°C.

Fish were humanely euthanized and brains taken for RNA extraction. The brains were snap frozen in liquid nitrogen and kept at -70°C until RNA extraction.

### Cloning of *S. aurata *myostatin-2 (saMSTN-2) promoter

Genomic DNA was isolated from fin clips using the protocol described earlier [30]. Two *S. aurata *fish were used for cloning the promoter: fish #17G which was found, following sequencing, to display two alleles of the promoter, named by us 'a' and 'b' (see Results section) and fish #0661 (sire of cross 1) which was found to be a heterozygote following the progeny analysis (see Results section), having the 'b' allele and a third allele, named by us 'c'. The sa*MSTN-2 *promoter was isolated from genomic DNA, digested with several restriction enzymes, using the linker-mediated PCR method [31]. The protocol used was similar to that reported earlier by us for cloning *S. aurata *fast skeletal myosin light chain-2 promoter (*saMLC2f*) [32], with minor changes. The nested PCR was performed by using the two linker-specific primers (L1 and L2) and two gene-specific primers (MSTNb-1 and MSTNb-2). The gene-specific primers were designed based on the nucleotide sequence of *S. aurata MSTN-2 *cDNA [GenBank accession number AY046314; [24]], complementary to the 5' end of the cDNA. The primers used in the study reported here are listed in Additional file 1. The amplified fragments were analyzed by electrophoresis on 1% agarose gels, purified by QIAquick Gel Extraction Kit (Qiagen GmbH, Hilden, Germany) and cloned in pGEM-T Easy vector (Promega, Madison, VI, USA). Plasmid DNA was purified by the QIAprep Spin Miniprep Kit (Qiagen GmbH) and sequenced. Comparison between the new sequences with that of *MSTN-2 *cDNA from *S. aurata *[24] confirmed their identity as being derived from the *MSTN-2 *gene.

### Cloning of *S. aurata *myostatin-2 (saMSTN-2) full length gene

The full-length of sa*MSTN-2 *gene sequence was amplified by PCR, using genomic DNA from fins of three *S. aurata *individuals: fish #17G (found following sequencing to display two alleles of the first intron, named by us 1R and 2R), fish #33 (found following sequencing also to possess allele 2R), and fish #13RD (found following sequencing to display a third allele of the first intron, named by us 4R). The primers MSTNb-2fw and MSTNb-7 (Additional file 1), were designed based on the published nucleotide sequence of sa*MSTN-2 *mRNA. The target sequence corresponds to the region extending 10 bp upstream from the translation start codon to 21 bp downstream of the stop codon. PCR reactions were performed using 3.75 Units of a proofreading Taq DNA polymerase (Expand Long Template PCR System; Roche Applied Science, Indianapolis, IN, USA) in 1X Buffer 2, 0.3 μM of each primer, and 0.35 mM of each nucleotide. After an initial denaturation step (3 min, 94°C), amplification was performed for 35 cycles (one cycle: 25 sec at 94°C, 45 sec at 61°C, 3 min at 68°C) followed by a final elongation period of 10 min at 68°C. The amplified fragments were analyzed by electrophoresis on 1% agarose gel and purified by QIAquick Gel Extraction Kit. To facilitate "T/A cloning" into pGEM-T Easy vector, the PCR products were polyA-tailed according to the procedure outlined in the pGEM-T Easy Technical Manual and cloned in pGEM-T Easy vector. Plasmid DNA was purified as above and was first sequenced with primers SP6 and T7. Additional primers were subsequently designed, according to the resulting sequences, in order to obtain the full-length sequence of the cloned gene: exon1-248fw, intron1-501fw, exon2-56fw, exon2-187rev (Additional file 1). The donor and acceptor splice sites were identified by comparison of the genomic sequences with the published cDNA sequence as well as our own mRNA sequence obtained from *S. aurata *brain (data not shown).

### Identification of transcription start site by rapid amplification of cDNA ends (RACE)

The transcription start site was identified with the FirstChoice^® ^RLM-RACE kit (Ambion, Inc. Austin, TX, USA), following the manufacturer's protocol, using total RNA (5 μg) from *S. aurata *brain, 5'RACE adaptor (Ambion) and random hexamers for reverse transcription. Primers for this 5'RACE nested PCR (MSTN2-exon2-261rev and MSTN2-exon2-187rev, Additional file 1) were designed based on the *MSTN-2 *cDNA sequence. The 600 bp amplified fragment was gel-purified, and sequenced.

### Analysis of saMSTN-2 promoter polymorphism

Analysis of promoter polymorphism was carried out by PCR amplifications of genomic DNAs from the collections described above using a reverse primer complementary to the 5'end of *MSTN-2 *coding region (MSTNb-1), which is in a region identical in all alleles. The forward primers were specific to each allele of *MSTN-2 *promoter, designed according to the sequences obtained from the cloned promoter fragments. The sequences of the primers are shown in Additional file 1. PCR reactions were performed using 1.25 Units of Taq DNA polymerase (New England Biolabs, Beverly, MA, USA) in 1× ThermoPol reaction Buffer, 0.5 μM of each primer, and 0.2 mM of each nucleotide. After an initial denaturation step (3 min, 95°C), amplification was performed for 30 cycles (one cycle: 45 sec at 95°C, 45 sec at 55°C, 2.5 min at 72°C) followed by a final elongation period of 10 min at 72°C. Amplification and length polymorphism of the various alleles at the promoter locus were detected according to the relative electrophoretic mobility of the PCR products on 1% agarose gel. Allele 'b', amplified using the 'b'-specific primers pair MSTNb-1/MSTNb-10 migrated as 1.4 kb. Allele 'c', amplified using the 'c'-specific primers pair MSTNb-1/MSTNb-13 migrated as 1.7 kb. Alleles 'a' and 'as', amplified using the 'a'-specific primers pair MSTNb-1/MSTNb-11 migrated as 1.9 and 1.7 kb, respectively.

### Analysis of saMSTN-2 intron1 polymorphism

Genomic DNAs from the five different collections (see above) were also used for analysis of intron 1 polymorphism by exon-primed intron-crossing (EPIC)-polymerase chain reaction (PCR) [33]. A pair of intron-spanning primers was designed in flanking exons based on the nucleotide sequence of sa*MSTN-2 *gene: MSTN-2-exon1-248fw and MSTN-2-exon2-187rev (Additional file 1). The amplified fragment included in addition to *MSTN-2 *intron 1 also 79 bp of exon 1 and 187 bp of exon 2. PCR reactions were performed using 1.25 Units of Taq DNA polymerase in 1× ThermoPol reaction Buffer, 0.5 μM of each primer, and 0.2 mM of each nucleotide. After an initial denaturation step (3 min, 95°C), amplification was performed for 35 cycles (one cycle: 45 sec at 94°C, 45 sec at 60°C, 2 min at 72°C) followed by a final elongation period of 10 min at 72°C. Length polymorphism of the various alleles at the intron 1 locus was detected according to the relative electrophoretic mobility of the PCR products on 1% agarose gel. Alleles 1R, 2R and 4R migrated as 1050, 1200 and 1500 bp, respectively. 1R and 2R refer to the presence of one or two copies of a 150 bp repeat at the 5'-end of the intron.

### Sequence analysis

The gene sequence, including repeat sequences, was analyzed both manually and by using BLASTN and nr/nt database of NCBI [34]. A multiple-sequence pre-alignment was performed using ClustalW [http://www.ebi.ac.uk/clustalw/,[35]], and refined by hand. The DNA sequence of the promoter and gene were analyzed with Gene Runner, Version 3.05 (Copyright ^© ^1994 Hastings software Inc) for restriction sites, with the TESS (Transcription Element Search Software on the WWW) program [http://www.cbil.upenn.edu/cgi-bin/tess/tess, [36]] for presence of transcription factors and with the RepeatMasker program for the presence of transposable elements http://www.repeatmasker.org.

### Data analysis

Allele frequencies, number of effective alleles, observed heterozygosity, expected heterozygosity, and inbreeding coefficient as well as χ^2 ^tests for deviation from Hardy-Weinberg equilibrium were calculated by AMOVA (Analysis of Molecular Variance) using GenAlEx 6.1 [37]. Mendelian inheritance of the two DNA markers in progeny of a full-sib family was determined using χ^2 ^test. UPGMA Cluster was done by using Nei [38] genetic distance and the TFPGA version 1.3 program [39] (1000 dememorization steps; 10 batches; 2000 permutations per batch).

## Results

### Isolation of saMSTN-2 gene upstream sequences (saMSTN-2 promoter)

**Figure 1:**
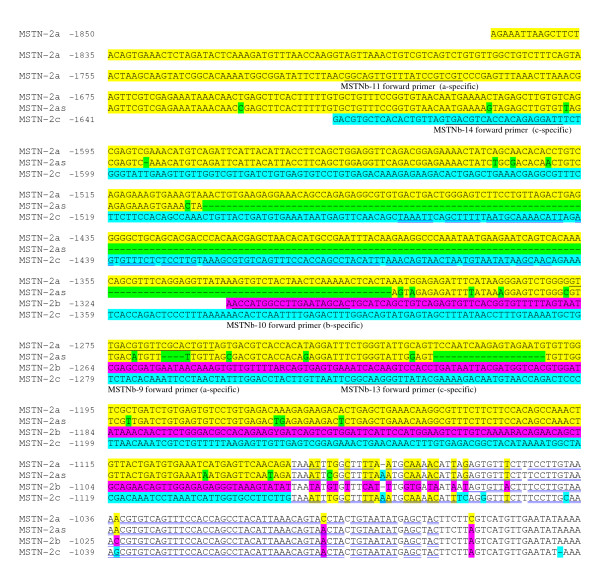
**Nucleotide sequence alignment of sa*MSTN-2 *promoter alleles (distal promoter)**. Sequences for sa*MSTN-2b *and *-2a *alleles are consensus sequences of 10 different clones of various length at the 5' end, obtained from fish #17G genomic DNA. Partial sequence for sa*MSTN-2as *is a consensus sequence of amplified fragment of two genomic DNAs, #8C and #13C, from the fifth DNA collection, which was obtained by PCR using the 'a'-specific forward primer MSTNb-11 and reverse primer MSTNb-1 and sequenced with primer MSTNb-11. Sequence for sa*MSTN-2c *is the consensus sequence from two clones obtained from amplified genomic DNA of fish #13 from the Ardag collection using the 'c'-specific forward primer MSTNb-14 and reverse primer MSTNb-7, cloned in pGEM-Teasy vector and sequenced with primers T7/SP6, MSTNb-3 and MSTNb-1. The first 19 nucleotides of allele 'c' derived from the cloned fragment of genomic DNA of fish #0661 (sire of cross1) (see Materials and Methods). Sequences corresponding to primers are underlined with single black line and the names are indicated underneath. Repeated sequences are double-underlined in blue. Gaps (-) are indicated in the individual sequences. Sequences highlighted in color are non-identical sequences between a, b, c, and a_s _alleles (yellow, pink, turquoise and green, respectively). Numbers on the left indicate distance from position +1 which has been assigned to the first nucleotide of the translation start codon ATG. GenBank Accession numbers of *MSTN-2a*, *-2b*, *-2as *and *-2c *are GQ379809, GQ379810, GQ379811, and HQ380026, respectively.

**Figure 2:**
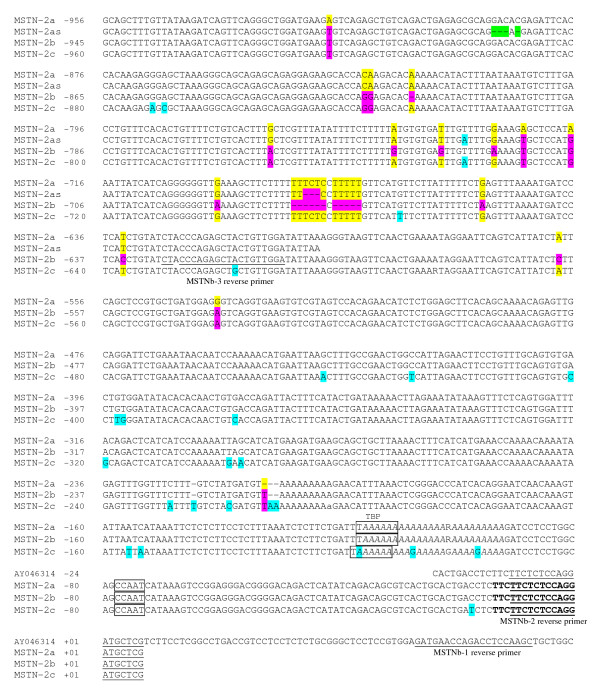
**Nucleotide sequence alignment of sa*MSTN-2 *promoter alleles (proximal promoter)**. Details are as in Figure 1. Position +1 has been assigned to the first nucleotide of the translation start codon ATG. The sequence in italics is the consensus sequence for the polymorphic mononucleotide repeat: the first mononucleotide A repeat (from -93 to -101) varies from 8 to 11 bp, and the second A mononucleotide repeat (from -103 to -116) varies from 14 to 23 bp. Sequences highlighted in color are non-identical sequences between a, b, c, and a_s _alleles (yellow, pink, turquoise and green, respectively). In bold letters is the sequence obtained by 5'RACE using brain RNA. TATA and CCAAT boxes are boxed. GenBank Accession numbers of *MSTN-2a*, *-2b*, *-2as *and *-2c *are GQ379809, GQ379810, GQ379811, and HQ380026, respectively.

Ten genomic fragments, containing the 5' flanking region of sa*MSTN-2 *gene, were isolated using genomic DNA from a single fish (#17G) digested with various restriction enzymes using the linker-mediated method (detailed in Materials and Methods). The genomic fragments ranged from 131 bp to 1857 bp in length. All fragments started 7 bp downstream of the translation start codon ATG of sa*MSTN-2 *gene (corresponding to primer MSTNb-2), and extended to the 5' flanking region. In few cases, more than one fragment was amplified from the same digestion; probably due to the presence of additional genomic DNA fragments cleaved at sites other than the specific restriction site. Comparison of the new sequences with that of sa*MSTN-2 *cDNA [[24], AY046314] confirmed their identity as being derived from the *MSTN-2 *gene (31 bp identity: from 7 bp downstream to 24 bp upstream of the ATG translation start codon). The presence of an identical 3' end (1050 bp long) in all fragments indicated that they were amplified from the same gene. A poly(A) repeat of 17 to 27 nt was found at the proximal promoter, approximately at position -117. Two potential binding sites for transcription factors, important for basal gene transcription, flank this repeat: immediately upstream, a binding site for the TATA binding protein (TBP) and downstream a CAAT box (details of the sequence analysis related to location of transcription factors as well as promoter activity of the various cloned fragments are included in a separate paper). Surprisingly, comparison between the sequences from the ten genomic fragments revealed close identity within the first 1050 bp located 5' to the first ATG codon but divergence of sequences located further upstream into two groups (Figure 1, Figure 2). This result suggested that there are at least two alleles of the *MSTN-2 *promoter, named by us *MSTN-2a *and *MSTN-2b*, which are present in the genomic DNA used for cloning the promoter. In addition to this major difference between the two alleles, many small differences (Single Nucleotide Polymorphism, SNPs) were found (Additional file 2 and sequences comparison in Figure 1 and Figure 2). As explained below, later on we identified an additional allele, *MSTN-2c*, which is included as well in Figure 1, Figure 2 and in Additional file 2.

**Figure 3:**
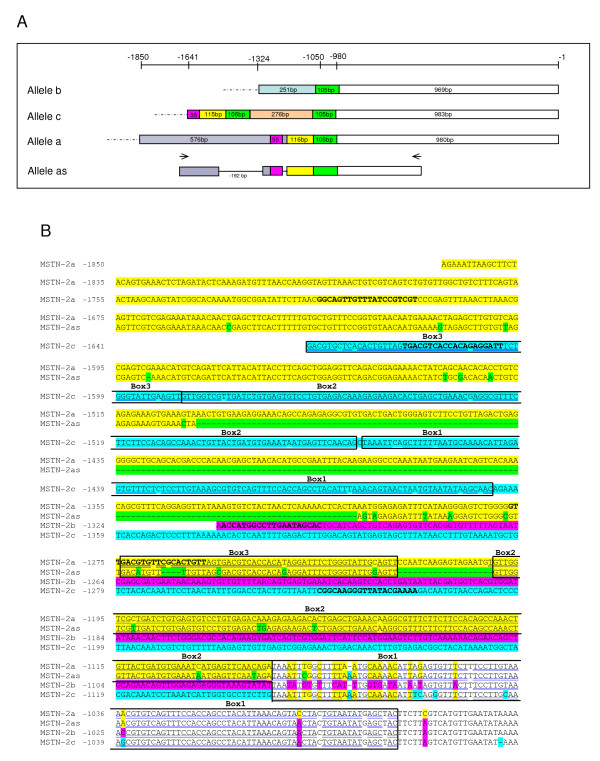
**Repeated sequences in sa*MSTN-2 *promoter alleles**. (A) Schematic presentation of repeated sequence elements in sa*MSTN-*2 promoter alleles. Box1 (green square) corresponds to sequences present twice in allele 'c' and once in alleles 'a' and 'b'. Box2 (yellow square) and Box3 (pink square) correspond to sequences present once in both allele 'c' and allele 'a'. Deletions are indicated by horizontal lines. Identical sequences in all alleles are shown by empty squares. 'b' specific sequences (pale blue square), 'c' specific sequences (peach square), 'a' specific sequences (grey square). Position -1 corresponds to the first nucleotide preceding the translation start codon ATG. (B) Alignment of repeated sequence elements in sa*MSTN-2 *promoter alleles. Included are regions upstream of nucleotide -946 (numbering is according allele 'b'), the same as that shown in Figure 1. Repeated sequences are boxed. Primer sequences are in bold letters. Other details are as in Figure 1.

In order to confirm the existence of two different alleles of the sa*MSTN-2 *promoter, genomic DNA from several *S. aurata *DNA collections (see Materials and Methods for details) were screened for the presence of these alleles: PCR amplifications were carried out using a reverse primer complementary to the 5'end of the *MSTN-2 *coding region (MSTNb-1) which is in a region identical in both alleles and forward allele-specific primers (MSTNb-10 and MSTNb-11, see Materials and Methods for details and Figure 1 and Figure 2 for primers' location). The results confirmed that the sa*MSTN-2 *promoter is polymorphic: amplification was observed in most individuals when the *MSTN-2b *specific primer was used (158 individuals out of 169), whereas only few individuals showed amplification when *MSTN-2a *specific primer was used (24 individuals out of 169). In some individuals amplification was seen with both sets of primers, while in others amplification was obtained with only one set but not with the other, indicating that these are two different alleles of sa*MSTN-2 *promoter, and not two different genes. The reasoning being that if these two forms represented two genes, one would expect to obtain amplification with both sets of primers for all the individuals. Moreover, the results indicate that the *MSTN-2a *allele is a rare allele, as it is poorly represented in the populations studied. Furthermore, in 6 out of the 24 individuals showing amplified fragments using the *MSTN-2a *specific primer, a shorter fragment than the expected one was obtained. Partial sequence analysis of this fragment confirmed its homology to *MSTN-2a *allele, but this "short" *MSTN-2a *allele (*MSTN-2as*) had in addition to SNPs and small deletions (Additional file 2), also a major deletion of 192 nucleotides between -1308 and -1499 of sa*MSTN-2a *(Figure 1 and Figure 3). Unexpectedly, in 6 individuals no amplification was obtained with either of the two allele-specific primers pairs, suggesting the existence of yet another variant of sa*MSTN-2 *promoter, with an unknown sequence.

### Allele segregation analysis of the saMSTN-2 promoter region: evidence for the presence of an additional allele

In order to confirm that the alleles of sa*MSTN-2 *5' flanking region were of the same gene and did not originate in two different genes (paralogs), and also to test for the mode of inheritance, the segregation of sa*MSTN-2 *alleles 'a' and 'b' was determined in *S. aurata *progeny of a full-sib family. The three available crosses were tested for the presence of *MSTN-2a *and/or *MSTN-2b *allele by PCR analysis using the allele-specific primer pairs as described above. Only the parents of Cross 1 proved to have different alleles of the *MSTN-2 *promoter whereas the parents of the two other crosses were homozygotes for allele 'b'. In Cross 1 the dam was a heterozygote (a/b) while the sire was b/b (although later on we found that it possesses a third allele 'c', see below). According to the Mendelian mode of inheritance, the expected allele segregation of one gene with two alleles in the progeny of parental genotypes as described above, is 50% a/b and 50% b/b. However, the results were different: only 11% (3 out of 26 individuals) showed amplification with both 'a'-specific and 'b'-specific primers sets, while 58% (15 out of 26 individuals) showed amplification only with 'b'- specific primers. Surprisingly, 31% (8 out of 26 individuals) showed amplification only with the 'a'-specific primers, although no a/a progeny was expected. However, if we hypothesize that the sire is heterozygote for *MSTN-2 *promoter and carries, in addition to one copy of *MSTN-2b *allele, another variant of *MSTN-2 *promoter (that we called *MSTN-2c*), not recognized by 'a'- and 'b'-specific sets of primers, then the expected genotypes for one gene with three alleles are a/b, b/b, a/c and b/c. In this case, with primers only for 'a' and 'b' alleles, genotypes b/b and b/c cannot be differentiated and a/c would be seen as 'a' only.

### Cloning of a third variant of the saMSTN-2 promoter

The third variant (allele 'c') of the sa*MSTN-2 *promoter was cloned using genomic DNA from the sire of Cross 1 (see above), which was suspected to be a b/c heterozygote. Genomic fragments of various sizes, containing the 5' flanking region of sa*MSTN-2 *gene, were obtained using the linker-mediated method (detailed in Materials and Methods). Fragments that were longer than ~1.3 kb were tested by PCR amplification using the 'b'-specific pair of primers to determine if they were derived from the 'b' allele, in order to be eliminated. One fragment of ~1.6 kb did not yield an amplification product, suggesting that it was derived from allele 'c'. This fragment was isolated, cloned and sequenced. Comparison of this sequence with those of *MSTN-2a *and *MSTN-2b *promoter alleles confirmed the existence of a new *MSTN-2 *promoter allele: *MSTN-2c *(Figure 1 and Figure 2). The sequence of the first 1050 bp upstream of the translation start codon ATG of allele 'c' is almost identical to alleles 'a' and 'b'; between nucleotides -1050 and -1088 the sequence is almost the same as allele 'a' but different from that of allele 'b'. Upstream of nucleotide -1088 the sequences differ completely (Figure 1). In addition, many SNPs were found between *MSTN-2c *and the two other alleles, probably in part due to the fact that the DNA used for cloning allele 'c' originated from an individual of a different collection than the one used for cloning *MSTN-2a *and *MSTN-2b *(Additional file 2, Figure 1 and Figure 2).

Careful sequence analysis of the region upstream of the first 1050 bp in the three alleles showed that several elements appear to be repeated in some or all sequences, at different positions (see Figure 3A for a schematic illustration and Figure 3B for sequences comparisons). The first identified element, box 1, is 105 bp long; it is present in all the alleles and appears twice in *MSTN-2c *allele. The two boxes are separated by 276 bp, which represent allele 'c'-specific sequences, showing no homology to any other region or to any other allele. Upstream of box 1, we identified two boxes, box 2 and box 3, that in allele 'c' are juxtaposed whereas in allele 'a' are separated by a 19 bp long element, representing an 'a'-specific sequence. Box 1 in allele 'b' and box 3 in allele 'a' are preceded by 'b'- or 'a'-specific sequences.

### Analysis of allele segregation in the saMSTN-2 promoter

In light of the discovery of a third promoter variant, *MSTN-2c*, cloned from the sire of cross 1, the presence of the *MSTN-2c *allele in the sire and progeny was determined by PCR amplification of genomic DNA using 'c'-specific primers set (see Materials and Methods). The forward primer was located in the 'c'-specific region (outside the boxes). As expected, the use of allele 'c'-specific primers pair resulted in an amplified fragment both in the sire and in the progeny (Table 1). The expected allele segregation of one gene with three alleles in the progeny of parents that are heterozygotes a/b and b/c, considering a Mendelian inheritance, is a:b:c in a 1:2:1 ratio. The allele frequency in the full-sib progeny was 21%:60%:19% (Table 1). The calculated χ^2 ^value (1.962) falls between the critical values of 10% and 90% and therefore, the hypothesis of a Mendelian inheritance of one gene with three alleles is accepted.

However, when the genotypes segregation was analyzed, a significant difference (χ^2 ^= 11.846, *p *< 0.01) was found between the observed and expected genotype frequencies (Table 1).

### Cloning and molecular organization of saMSTN-2 full length gene

**Figure 4:**
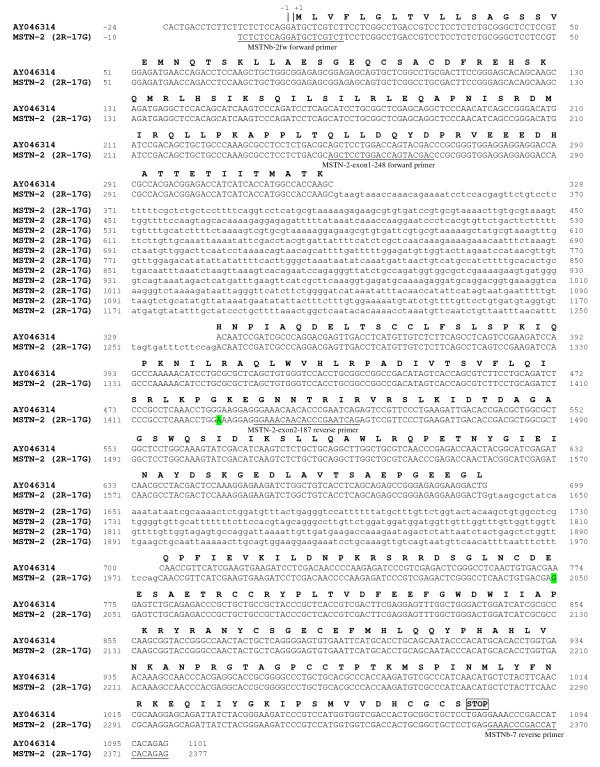
**Sequence analysis of sa*MSTN-2 *gene**. Comparison between sa*MSTN-2 *gene and the published sa*MSTN-2 *cDNA sequences. MSTN-2 (2R-17G) refers to the nucleotide sequence of sa*MSTN-2 *gene based on 2R-17G allele. Deduced amino acid sequence is shown in single-letter code. Sequences corresponding to primers are underlined and the names are indicated underneath. Exons are in uppercase and introns in lowercase letters. Exon1: 328 bp; Intron1: 938 bp; Exon2: 371 bp; Intron2: 338 bp; Exon3: 381 bp (until stop codon TGA); +1 refers to the first nucleotide of the translation start codon. The two nucleotides that are different from the published sequence are highlighted. GenBank accession number for sa*MSTN-2 *gene sequence (MSTN-2 variant 2R-17G) is GQ379805. AY046314, *MSTN-2 *cDNA sequence [24].

The sa*MSTN-2 *gene, amplified from genomic DNA using primers MSTNb-2fw and MSTNb-7, spans about 2.4 kb. The sequence and primer locations are illustrated in Figure 4. The gene has three exons encoding an open reading frame of 1077 bp, which is translated into a 358 amino acid long preproMSTN-2. The three exons are interrupted by two introns, both with consensus 5' and 3' intron splice sites (Figure 4).

Surprisingly, analysis of the resulting PCR products revealed a doublet of approximately 2.3-2.5 kb for DNA #17G (see Materials and Methods) and only one band of a similar size as the upper band of DNA #17G, for DNA #33, suggesting the existence of at least two alleles of the *MSTN-2 *gene. Since in DNA #33 only one fragment was amplified, we predicted that these two fragments do not represent two paralogs but are rather two alleles of the same gene, which are present as a homozygote in fish #33 and as a heterozygote in fish #17G. Sequence analysis of the three fragments: 1R-17G (the lower fragment from #17G), 2R-17G (the upper fragment from #17G) and the fragment from #33 (2R-33), confirmed that 2R-33 was almost identical to 2R-17G (except for SNPs, see Additional file 3). Moreover, comparison of the three genomic fragments to previously determined *MSTN-2 *cDNA sequences confirmed their identity as being derived from the *MSTN-2 *gene.

**Figure 5:**
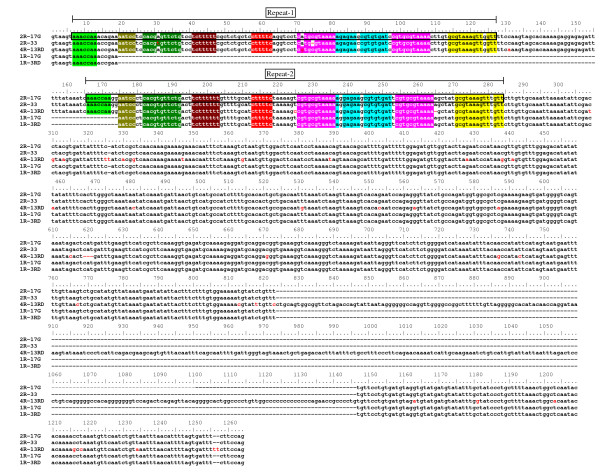
**Nucleotide sequence alignment of sa*MSTN-2 *intron1 alleles**. Alignment of nucleotide sequence of sa*MSTN-2 *intron 1 alleles 1R, 2R and 4R from several individuals (as detailed in Materials and Methods). Repeat-1 and Repeat-2 are boxed and also indicated above the sequence. Identical sequences are highlighted in various colors. SNPs are indicated in red letters. Deletions are indicated by (-).GenBank Accession numbers of *MSTN-2 *variants 2R-17G, 2R-33, 4R-13RD, 1R-17G and 1R-3RD are GQ379805, GQ379806, HQ380027, GQ379807 and HQ380028, respectively.

The size difference observed between the amplified genomic fragments is due to differences in the length of intron 1 which is 783 bp in 1R-17G, while 2R-17G is 938 bp long (937 bp in 2R-33) (Figure 5). Sequence analysis of the two alleles revealed that the length difference is due to the presence of one or two copies of a repeated block of approximately 150 bp located at the 5'end of intron 1 (Figure 5). The two repeats in 2R-17G and 2R-33 show 73% identity whereas the repeat present in 1R-17G shows a higher identity (93%) to the second repeat in both 2R-17G and 2R-33 than to the first repeat in 2R-17G and 2R-33 (only 80% identity).

The transcriptional start site of the sa*MSTN-2 *gene was determined using the 5' RACE technique. A transcription initiation region was identified (Figure 2) at position -14 from the translation start codon. This site is located 10-nucleotides downstream of the 5' end of the previously reported sea bream *MSTN-2 *cDNA [24].

### The saMSTN-2 gene is polymorphic in length: identification of a third allele of intron 1

**Figure 6:**
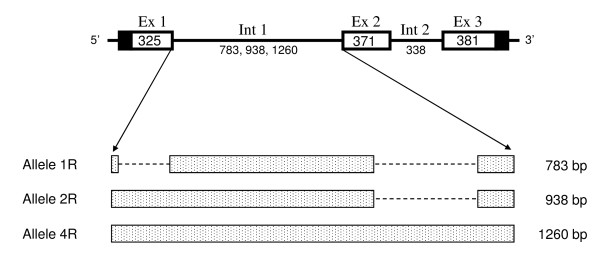
**Scheme of sa*MSTN-2 *gene and the three alleles of intron 1 (1R, 2R, 4R)**. Ex, exon; Int, intron. Numbers indicate length in bp of the exons and the introns.

In order to determine if the intronic alleles are rare or common, several *S. aurata *DNA collections (the same as those used for analysis of the promoter polymorphism, see above) were tested for the presence of these alleles, using EPIC-PCR (see Materials and Methods). Surprisingly, in addition to the two expected alleles, an additional fragment was amplified, migrating at a higher molecular weight. This fragment appeared either alone (homozygote state) or together with one of the expected bands for 1R or 2R alleles (heterozygote state). The new allele was termed 4R since we predicted that it might contain four repeats of the 150 bp unit. However, cloning and sequence analysis of the 4R-PCR fragment from three independent DNA samples revealed the existence of a 323 bp insertion, located 116 bp upstream of the 3' end of sa*MSTN-2 *intron 1, and not at the 5' end of the intron, where the repeats are located (see scheme in Figure 6). No identity was found between this 4R-specific insertion and the repeated sequences found at the 5' end of *MSTN-2 *intron 1. In addition to this major difference, several minor differences (SNPs) were observed between 1R, 2R and 4R alleles (Additional file 3). In order to compare the entire gene sequence containing the 4R allele to the two other alleles, the region from 10 bp upstream of the translation start codon to 21 bp downstream of the stop codon was amplified (using the same primers as those used for cloning the gene containing 1R and 2R alleles, as described above), cloned and sequenced. We confirmed that no major differences exist between the 4R and the 1R and 2R alleles, except for the previously identified insertion at the 3' end of sa*MSTN-2 *intron 1, but many SNPs (small deletions and small additions) were found (see Additional file 3). Furthermore, PCR amplification of genomic DNA from two fish carrying two alleles combinations - alleles 'a' and 1R or allele 'c' and 4R - using a forward primer located at the promoter and a reverse primer located at the 3' UTR of the gene, verified that indeed these alleles originated from one single gene.

### Allele segregation analysis of the saMSTN-2 intron 1

In order to confirm that the alleles of intron 1 belong to the same gene and are also inherited in a Mendelian way, the segregation of sa*MSTN-2 *intronic alleles was determined by EPIC-PCR (see Materials and Methods). As observed above for the promoter alleles, only parents of Cross 1 were heterozygotes for intron 1 of *MSTN-2*: the dam was 1R/2R and the sire 2R/4R (Table 1). The two other crosses were homozygotes for allele 2R and therefore were not included in the analysis. Genotypes of *MSTN-2 *intron1 were determined in 27 individuals of Cross 1 full-sib progeny. The expected allele segregation of the progeny for one gene with three alleles is 1R:2R:4R with a 1:2:1 ratio (Table 1). The calculated χ^2 ^value (1.999) for the observed allele frequency falls between the critical values of 10% and 90% (Table 1). Therefore, the hypothesis that the observed allele frequencies are as predicted by Mendelian inheritance for three alleles of one gene was accepted.

However, when the genotype segregation was analyzed, a significant difference was found between the observed and expected genotype frequencies. The results obtained were as follow: 1R/2R, n = 4; 2R/2R, n = 13; 1R/4R, n = 8; 2R/4R, n = 2 (Table 1). The numbers differ significantly from the expected 6.75 in each genotype class (χ^2 ^= 10.481, *p *< 0.025).

### Analysis of saMSTN-2 promoter and intron 1 polymorphism and relation to fish size or fish origin

The frequency of sa*MSTN-2c *allele in *S. aurata *populations was determined using the same DNA collections that were screened earlier for the presence of sa*MSTN-2a *and sa*MSTN-2b *alleles (see above), and a similar strategy (allele 'c'-specific primers pair). Genotype and allele frequency data for the promoter and intron 1 alleles are summarized in Table 2. A χ^2 ^test analysis for both loci showed that the populations analyzed conform to Hardy-Weinberg equilibrium, suggesting that they represent panmictic groups.

**Table 2:** Allele and genotype frequencies for the two polymorphic loci of sa*MSTN-2 *gene in different DNA collections

			Atlit hatchery			Ardag hatchery			Portugal hatchery	G/C	Italy (wild)
							
			Large	Average	Total	Large	Small	Total			
**ALLELE** **FREQUENCIES**	Intron1	1R	0.056	0.040	0.048	0.017	0.000	0.008	0.000	0.067	0.065
		2R	0.759	0.780	0.769	0.517	0.500	0.508	0.842	0.733	0.761
		4R	0.185	0.180	0.183	0.467	0.500	0.483	0.158	0.200	0.174
		N	27	25	52	30	30	60	19	15	23
	
	Promoter	a	0.074	0.140	0.106	0.017	0.000	0.008	0.000	0.067	0.087
		a_s_	0.019	0.040	0.029	0.000	0.000	0.000	0.000	0.067	0.022
		b	0.611	0.620	0.615	0.750	0.750	0.750	0.684	0.733	0.717
		c	0.296	0.200	0.250	0.233	0.250	0.242	0.316	0.133	0.174
		N	27	25	52	30	30	60	19	15	23

**GENOTYPE** **FREQUENCIES**	Intron1	1R/1R	0.000	0.000	0.000	0.000	0.000	0.000	0.000	0.000	0.000
		2R/2R	0.593	0.560	0.577	0.267	0.333	0.300	0.684	0.467	0.522
		4R/4R	0.037	0.000	0.019	0.233	0.333	0.283	0.000	0.000	0.000
		1R/2R	0.074	0.080	0.077	0.033	0.000	0.017	0.000	0.133	0.130
		2R/4R	0.259	0.360	0.308	0.467	0.333	0.400	0.316	0.400	0.348
		1R/4R	0.037	0.000	0.019	0.000	0.000	0.000	0.000	0.000	0.000
		N	27	25	52	30	30	60	19	15	23
	
	Promoter	a/a	0.000	0.000	0.000	0.000	0.000	0.000	0.000	0.000	0.000
		b/b	0.370	0.360	0.365	0.567	0.567	0.567	0.368	0.467	0.435
		c/c	0.074	0.000	0.038	0.067	0.067	0.067	0.000	0.000	0.000
		a/b	0.074	0.200	0.135	0.033	0.000	0.017	0.000	0.133	0.174
		b/c	0.370	0.280	0.327	0.333	0.367	0.350	0.632	0.267	0.348
		a/c	0.074	0.080	0.077	0.000	0.000	0.000	0.000	0.000	0.000
		a_s_/b	0.037	0.040	0.038	0.000	0.000	0.000	0.000	0.133	0.043
		a_s_/c	0.000	0.040	0.019	0.000	0.000	0.000	0.000	0.000	0.000
		N	27	25	52	30	30	60	19	15	23

In hatcheries from Atlit and Ardag two size groups were included in the analysis: large and average (Atlit); large and small (Ardag).

The results of the analysis of promoter allele frequencies in the various collections (detailed in Additional file 4) can be summarized as follow: (i) The *MSTN-2a *promoter allele appeared to be a rare allele (24 out of 338) compared to *MSTN-2b *and *MSTN-2c *alleles (235/338 and 79/338, respectively); (ii) The rare allele *MSTN-2a *was observed only in a heterozygous state; (iii) The *MSTN-2c *allele was also mainly found at a heterozygous state (6 homozygotes compared to 67 heterozygotes); (iv) The frequency of the 'a' allele was very low (0-0.8%) in Ardag and Portuguese collections. By contrast, Atlit, the wild population from Italy and the 'G/C' collections had much higher frequencies of the 'a' allele (13.5%, 10.9% and 13.4%, respectively); (v) The 'b' allele had a similar frequency in four of the DNA collections, except for Atlit; (vi) In two of the collections with a higher allele 'a' frequency, this increase was at the expense of the *MSTN-2c *allele frequency, which decreased; (vii) The frequencies of homozygotes and heterozygotes carrying *MSTN-2b *allele differed between the various DNA collections. In the Atlit and Portuguese collections we found fewer homozygotes than heterozygotes (19 and 7 homozygotes vs 26 and 12 heterozygotes, respectively) whereas in the Italian and the 'G/C' collections, homozygotes and heterozygotes were observed in the same proportions (10 and 7 homozygotes compared to 13 and 8 heterozygotes, respectively). On the other hand, in the partially domesticated Ardag stock, the ratio between homozygotes and heterozygotes was reversed (34 homozygotes and 22 heterozygotes).

Allele frequency analysis of sa*MSTN-2 *intron 1 in the five collections (detailed in additional file 5), showed the following: (i) The 1R allele appeared to be a much rarer allele (11/338) than the 4R allele (97/338) and the most commonly observed 2R allele (230/338); (ii) The rare allele 1R was observed only in a heterozygous state (one 1R/4R compared to 10 1R/2R); (iii) Overall, the 2R allele was observed at almost the same frequency in the heterozygous and homozygous state (70 and 80 fish, respectively). However, the proportion of heterozygotes and homozygotes differed amongst the five collections. In the Portuguese and Atlit collections, we found more homozygotes than heterozygotes (13 and 30 2R-homozygotes vs 6 and 20 2R-heterozygotes, respectively). In the 'G/C' and Italian collections, homozygotes and heterozygotes were observed at the same proportions (7 and 12 compared to 8 and 11) while in the partially domesticated Ardag stock the ratio between homozygotes and heterozygotes was reversed (18 2R-homozygotes and 25 2R-heterozygotes); (iv) The 4R allele was observed almost exclusively as a heterozygous state in four of our collections (only one 4R-homozygote vs 37 4R-heterozygotes). By contrast, in the Ardag hatchery a substantial proportion of 4R-homozygotes were found (17 4R-homozygotes vs 24 4R-heterozygotes). (v) The frequency of the 1R allele was very low (0-0.8%) in the Ardag and Portuguese collections whereas in the Atlit, Italian and 'G/C' collections a higher frequency was found (4.8%, 6.5% and 6.7%, respectively). By contrast, the 2R and 4R alleles showed a slightly different pattern. Whereas all collections except for Ardag had similar frequencies of the 2R allele (73%-84%), the Ardag population had 51%. With respect to the 4R allele, the frequency in Ardag was 48% while in all other collections it ranged between 16% and 20% (additional file 5).

The analysis of heterozygosity of both polymorphic loci in all five populations is summarized in Table 3. Observed heterozygosity was slightly higher than expected heterozygosity in all populations except RD (Ardag). The latter was the only population with a positive value of *F*is, indicating deficiency in heterozygotes and inbreeding, whereas in all other collections negative values were found for *F*is, indicating outbreeding and random mating. The observation that the various alleles were unevenly represented in the populations, with few being rare, agrees with the calculated effective allele number being lower than the actual number of alleles (Table 3).

**Table 3:** Variability of the two loci in the sa*MSTN-2 *gene in five DNAc ollections of *Sparus aurata*

Locus	Population	*N*	***N***_**A**_	***N***_**E**_	***H***_**O**_	***H***_**E**_	***F***_**IS**_	*P*-value
Intron1	RD	60	3	2.032	0.417	0.508	0.180	0.389
	AT	52	3	1.594	0.404	0.373	-0.084	0.897
	G/C	15	3	1.718	0.533	0.418	-0.277	0.576
	ITAL	23	3	1.630	0.478	0.387	-0.237	0.518
	PORT	19	2	1.362	0.316	0.266	-0.187	0.414

Promoter	RD	60	3	1.610	0.367	0.379	0.033	0.935
	AT	52	4	2.206	0.596	0.547	-0.090	0.902
	G/C	15	4	1.772	0.533	0.436	-0.224	0.921
	ITAL	23	4	1.809	0.565	0.447	-0.264	0.735
	PORT	19	2	1.761	0.632	0.432	-0.462	0.044*

RD, Ardag hatchery; AT, Atlit hatchery; 'G/C' Mevo'ot Yam School, Michmoret; ITAL, wild population from Italy; PORT, INIP hatchery, Portugal; *N*, number of individuals; *N*_A_, number of alleles; *N*_E_, number of effective alleles; *H*_O _and *H*_E_, observed and expected heterozygosities; *F*_IS_, inbreeding coefficient. Parameters were obtained by AMOVA (Analysis of Molecular Variance) using GenAlEx program for codominant data. P-value for departure from Hardy Weinberg equilibrium (HWE) was obtained from χ^2 ^test calculated using also GenAlEx program.*significant deviation from HWE.

To determine whether there is an association between allele distribution and growth in a hatchery population, we analyzed the genotype and allele frequencies distribution between two size groups for the two Israeli hatcheries. The data is included in additional file 4 and additional file 5. Such an evaluation revealed no significant difference in the frequency of *MSTN-2 *promoter alleles between large and small fish in the Ardag hatchery. By contrast, in the Atlit hatchery "large" fish showed a lower frequency of allele 'a' and a higher frequency of allele 'c' compared to the "average" body weight group (9.3% vs 18% for allele 'a' and 29.6% vs 20% for allele 'c'). No difference in allele frequency between the two size groups in the two hatcheries was seen with respect to *MSTN-2 *intron 1 (Additional file 5).

Heterozygosity calculated separately for the two size-selected groups in the two commercial hatcheries is summarized in Table 4. Values of observed heterozygosity were slightly higher than expected in Atlit samples (ATL and ATM) while in Ardag (RDS and RDL) values of observed heterozygosity were slightly lower than expected and the inbreeding coefficient was positive, suggesting deficiency of heterozygosity in Ardag hatchery.

**Table 4:** Variability of the two loci in the sa*MSTN-2 *gene of two size groups from two *S. aurata *hatcheries

Locus	Population	N	***N***_**A**_	***N***_**E**_	***H***_**O**_	***H***_**E**_	***F***_***IS***_	*P-*value
Intron-1	ATL	27	3	1.629	0.370	0.386	0.041	0.911
	ATM	25	3	1.557	0.440	0.358	-0.230	0.575
	RDL	30	3	2.062	0.500	0.515	0.029	0.807
	RDS	30	2	2.000	0.333	0.500	0.333	0.068

Promoter	ATL	27	4	2.141	0.556	0.533	-0.042	0.961
	ATM	25	4	2.244	0.640	0.554	-0.154	0.776
	RDL	30	3	1.620	0.367	0.383	0.042	0.934
	RDS	30	2	1.600	0.367	0.375	0.022	0.903

RDS and RDL, small and large fish from Ardag hatchery; ATM and ATL, average and large fish from Atlit hatchery; *N*, number of individuals; *N*_A_, number of alleles; *N*_E_, number of effective alleles; *H*_O _and *H*_E_, observed and expected heterozygosities; *F*_IS_, inbreeding coefficient. Parameters were obtained by AMOVA (Analysis of Molecular Variance) using GenAlEx program for codominant data. P-value for departure from Hardy Weinberg equilibrium was obtained form χ^2 ^test calculated using also GenAlEx program.

**Figure 7:**
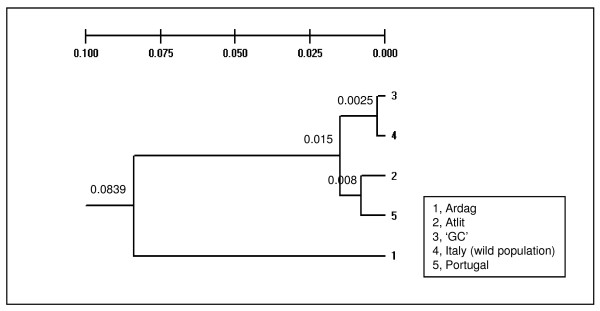
**UPGMA dendrogram based on Nei's genetic distance **[38]. Analysis was performed using the two DNA markers and five DNA collections with the TFPGA version 3.1 program. 1, Ardag; 2, Atlit; 3, 'GC'; 4, Italy (wild population); 5, Portugal. Numbers above certain branches show Nei's distance for the given node.

UPGMA Cluster analysis using Nei's genetic distances [38] for both polymorphic loci showed that the 'GC' fish and the wild population from Italy are similar, as are 'Atlit' and the fish from Portugal while all four are different from 'Ardag' (Figure 7).

## Discussion

In the present study we report on the identification of polymorphism of both the promoter and first intron of *MSTN-2 *gene in the marine fish, *S. aurata*. We found three alleles of the first intron and three alleles of the promoter. Sequences comparison of the alleles showed that polymorphism of the introns is due to the presence of one or two repeated blocks of 150 bp, resulting in the alleles 1R and 2R. The third allele, 4R, had an additional insertion of a DNA block of 323 bp. In the case of the promoter variation, the different alleles contain blocks of repeated sequences in addition to allele-specific sequences. We also found some SNPs and INDELs (gaps in sequences alignment), which could potentially reflect other alleles, but a more comprehensive study of a larger number of samples using PCR-SSCP would be required to confirm this possibility, and we hope to do this in a future study.

The allele polymorphism in the five populations that were analyzed in the current study is based on differential mobility of amplified DNA by gel electrophoresis. Several lines of evidence led to our conclusion that these alleles originated from the same gene and are not paralogs. First, analysis of allele segregation of the two DNA markers in a full-sib progeny showed that both are inherited in a Mendelian way. Second, analysis of individuals from several DNA collections (several cultured and one wild population) showed the existence of only one (homozygote state) or two variants (heterozygote state) for each of the DNA markers. If these were paralogs, one would expect to see all variants in each individual.

The presence of repeated blocks of DNA in the promoter and in the first intron of sa*MSTN-2 *gene suggests that both have been subjected to various chromosomal rearrangements during the course of evolution, reflecting events such as insertions, deletions or duplication, although we can only speculate on the mechanism. For example, an event of recombination between the two boxes 1 in allele 'c' of the promoter could have generated allele 'a'. The model suggested for co-initiation of intra-allelic duplication, conversion and crossover in human minisatellites [40], and the revised model for meiotic mutation events demonstrating the formation of intra-allelic events with duplications flanking the converted motifs [41], might explain the events leading to the generation of the 2R intronic allele from the 1R intronic allele. Generation of 4R intronic allele could be the result of breakpoints and translocation of a genomic DNA fragment from the osteocalcin gene to *MSTN-2 *gene or *vice versa *(see also below). Interestingly, a polymorphism of *MSTN *5' regulatory region as a result of an insertion of 386 bp was described recently in pigs [42].

The RepeatMasker program failed to find any transposable elements in sa*MSTN-2 *promoter variants or in sa*MSTN-2 *gene. A BLAST search using the various alleles of sa*MSTN-2 *promoter did not find any significant sequence similarity apart from the first 31 nucleotides of sa*MSTN-2 *mRNA. Similarly, a BLAST search using the full-length sa*MSTN-2 *gene sequence found similarity only with *GDF-8 *(*MSTN*) sequences, while a search using alleles 1R and 2R of intron 1 of sa*MSTN-2 *did not find any similar sequence, suggesting that the repeat region is unique to intron1 of sa*MSTN-2 *gene. Interestingly, BLAST search using the 4R allele of intron 1 showed a 92% identity between the region spanning nucleotide 822 to 1142 (4R-specific region) and *S. aurata *osteocalcin gene [Accession Number AF289506; [43]], from nucleotide 2655 to 2971, which corresponds to part of osteocalcin intron 2. This 320 bp identity between the two genes may reflect an event of duplication and translocation that occurred in *S. aurata *genome during the course of evolution. In the absence of chromosomal assignment to both genes, it is difficult to propose if this event was DNA shuffling between two chromosomes or within the same chromosome. Linkage analysis assigned osteocalcin to Radiation hybrid 24 together with growth hormone (GH) and prolactin [44] but no information is available on the *MSTN-2 *location. However, data from Ensembl shows that in both *Tetraodon *and zebrafish, *MSTN-2 *and osteocalcin genes are located on different chromosomes. Thus, in *Tetraodon MSTN-2 *is located on chr 3 and osteocalcin on chr 11 and in zebrafish *MSTN-2 *is on chr 22 and osteocalcin on chr 14. We have not yet identified upstream sequences that should be identical for all the promoter alleles. This will become possible only by cloning much longer genomic fragments than those obtained in the current study.

It should be emphasized that all the sequenced alleles of sa*MSTN-2 *first intron contain the consensus donor site gtaagt and acceptor site tttcttccag. The second intron also has consensus sites with donor gtaagc and acceptor tttctttccag. Nevertheless, although intron 1 of sa*MSTN-2 *has typical exon/intron splice site sequences, the BDGP (Berkeley Drosophila Genome Project) program http://www.fruitfly.org/seq_tools/splice.html predicted several additional splice sites within the intron 1 sequence. In particular, there is a predicted acceptor site with a higher score than the actual acceptor splicing site (0.99 compared to 0.98), located 65 bp downstream of intron 1's 5' end, and is present in the 2R allele but not in the 1R allele.

Earlier studies have shown that in *S. aurata *the *MSTN-2 *gene is expressed almost exclusively in the brain [24], although in juveniles Atlantic salmon *MSTN-2 *transcripts were detected in other tissues as well (red muscle, heart, intestine and ovary) when measured by RT-PCR [26]. Expression of the *MSTN-2 *gene in tissues other than brain was shown also in coho salmon [25], zebrafish [27] and rainbow trout [22]. The question of *MSTN-2 *functionality in brain or in other tissues is still unclear. Several reports suggested the possible involvement of MSTN-2 in fish growth. Over-expression of MSTN-2 in zebrafish [45] resulted in decreased dystrophin expression and muscle dystrophy. In another report [46] GH administration to zebrafish had an effect on MSTN-1 and MSTN-2 expression in white muscle. Finally, transgenic coho salmon over-expressing GH exhibited decreased levels of MSTN-2 expression in white muscle [25]. A recent study [22] reported that in rainbow trout, *MSTN-2a *mRNA is spliced into mature RNA only in the brain; in the gills both spliced and unspliced are found while in all other tissues tested *MSTN-2a *exists only as unspliced transcript. Data from our laboratory confirmed that also in *S. aurata*, *MSTN-2 *exists in brain as both spliced and unspliced transcripts while in muscle only unspliced transcripts were found (unpublished data). In one human case, a mutant *MSTN *has been described with an IVS1+5 g→a transition at the splice donor site in intron 1, causing a splice to occur 108 bp downstream at a cryptic splice site. This mutation produces a larger transcript in the mutant and results in a premature termination codon and gross muscle hypertrophy in a child [17]. Interestingly, our sequencing data of genomic DNA from several individuals indicates that in contrast to the findings in Atlantic salmon [26] and in rainbow trout [22], no stop codons were found in sa*MSTN-2 *gene, suggesting that this phenomenon of pseudogenes of *MSTN-2 *might be unique to salmonids and maybe related to whether the different fish species are tetraploid or not.

Previous studies in our laboratory have shown an extensive polymorphism of the *GH *gene in *S. aurata *due to various numbers of tandem repeats (minisatellites) in the first and third introns and a polymorphic microsatellite present in the promoter region [30,47]. Polymorphism of the first and third introns of the *GH *gene was found also in other species of the family Sparidae [48], pointing to the conclusion that GH genes in this family are highly polymorphic. Here we show that another gene, *MSTN-2*, is also polymorphic in *S*. *aurata *in both its first intron and promoter. The results of the present study have potential applications in aquaculture. The Mendelian inheritance implies that these two DNA markers can be useful for parentage determination. In particular, they can be useful for identifying the fish in the broodstock that participate in reproduction in a species with group spawning like *S. aurata *[49]. However, while the allele segregation showed a Mendelian inheritance, when genotypes were considered, a deviation from Mendelian inheritance was found and significant differences were found between the observed and expected genotype frequencies both for the promoter and for intron 1. These differences could be due to the relative small number of individuals tested. Alternatively, the discrepancy found between the expected and observed genotype frequencies can be due to the fact that these individuals were not sampled randomly but sampling was rather biased towards body weight and the largest and smallest of the progeny were chosen for analysis. This might indicate a link between fish size (growth rate) and certain alleles of *MSTN-2 *promoter or intron 1, making *MSTN-2 *a candidate gene for genetic selection for desirable traits. Another possibility for the differences seen between observed and expected genotype frequencies in the progeny might be the fact that the sire in Cross 1 is a carrier of the rare recessive mutation "ebony" which is lethal in homozygotes, but which is strongly heterotic for growth when fish are heterozygotes for this mutation [29].

Analysis of the five populations using the two polymorphic DNA loci showed no deviation from Hardy-Weinberg Equilibrium. The statistical analysis also showed positive values of *F*is for the population of Ardag hatchery, indicating deficiency of heterozygotes and inbreeding. This probably is the result of growth selection for several generations. Therefore, the two DNA markers employed in the current study can also be valuable in aquaculture industry to determine the degree of inbreeding in the cultured population. In addition, the analysis demonstrated that all the other (non-Ardag) populations studied are not different from each other.

Although the current study focused on major events of duplication, rearrangements and insertions of DNA sequences that resulted in polymorphism of both sa*MSTN-2 *promoter and intron 1, the degree of polymorphism is probably much higher as a result of many SNPs that were found in both the promoter and in the gene. Presence of SNPs has the potential of being applied to aquaculture in the future.

The development of genetic markers for marker-assisted selection in domestic animals has had a great impact on animal genetics and breeding. One of the candidate genes for such an approach is *MSTN *due to its critical role in muscle growth. SNPs in the *MSTN *gene were found to be positively correlated in chicken with production traits such as abdominal fat percentage, birth weight, breast muscle percentage and breast muscle weight [50]. Despite the large effects of *MSTN *mutations in different cattle breeds (as discussed above), no mutations with major effects have been described in pigs. Three point mutations at the promoter, intron 1 and exon 3 were reported in the porcine *MSTN *gene [51]. These authors also showed allele frequency differences between Chinese and Western breeds for at least two of these SNPs, but the differences may reflect the different origins of these breeds rather than any association with phenotypic traits. A different study [52] scanned for SNPs in the promoter, exon 2 and exon 3 of the *MSTN *gene in different Western pig breeds, and the only polymorphic site found was the same change at the exon 3 described earlier [53]. Statistical analysis did not reveal any differences in loin or ham meatiness between the pigs of the three genotypes. However, others have shown two SNPs in the *MSTN *promoter of pigs that were associated with growth and meat quality traits in two commercial populations [54] and SNPs in the promoter of the *MSTN *gene in Yorkshire pigs which showed an association with early growth traits [42]. In sheep, an association was founds between MSTN SNPs and growth and carcass traits [55].

In contrast to the vast research on application of polymorphism of *MSTN *to phenotypic traits in domestic animals, research into polymorphism of *MSTN *in fish is relatively scarce. A polymorphic microsatellite has been described in the 3' UTR of *MSTN *cDNA of shi drum, although no association with quantitative traits was determined [56]. Multiple microsatellites have been described in the promoter of the catfish *MSTN *gene: three microsatellites in intron 1 and one in intron 2 and in addition one microsatellite in the 3' UTR [57]. In addition, these authors described SNPs in various coding and non-coding regions but no attempts were made to study polymorphism of these microsatellites in the population or correlate the SNPs with quantitative traits. SNPs were also reported in a preliminary study in rainbow trout strains exhibiting distinct hyperplastic growth traits [58].

Phylogenetic analysis of gnathostome *GDF-11 *and *MSTN *sequences showed that tetrapods clustered separately from teleosts [59]. The teleost *MSTN *was separated into *MSTN-1 *and *MSTN-2*, consistent with the idea that after two rounds of genome duplications in the lineage leading to jawed vertebrates, the genome was duplicated a third time in the lineage leading to teleost fish [60,61]. Moreover, phylogenetic analysis and sequence comparisons suggested that legitimate *MSTN-2 *is present in the sea bream, shi drum, zebrafish, *Fugu *and rainbow trout. In the latter, two forms of *MSTN-2 *were identified: *MSTN-2a *and *MSTN-2b *[22]. In this context it should be noted that the previously identified Tmyostatin1 and Tmyostatin2 in trout [62], were re-classified as *MSTN-1a *and *MSTN-1b *[20]. Clustal analysis of the trout gene showed that the 5' and 3' ends of the two introns are almost identical in the two forms; however, one of the two forms was longer by ~ 500 bp as a result of several insertions [62]. A search in GenBank data base revealed two additional entries for *MSTN-2*: a genomic sequence of barramundi *Lates calacrifer MSTN-2 *(Accession number GU590863) and a genomic sequence for large yellow croaker *Larimichthys crocea MSTN-2 *(Accession number EU571244). Clustal comparison of the amino acid sequences of these two new *MSTN-2 *forms with that of sa*MSTN-2 *revealed 93-95% identity (342 out of 359 identical residues between sea bream and croaker), confirming their identity as *MSTN-2*. Despite this high conservation in amino acid sequence between these two species, the sequence of the first intron of the croaker *MSTN-2 *gene is very different from that of sa*MSTN-2*.

**Figure 8:**
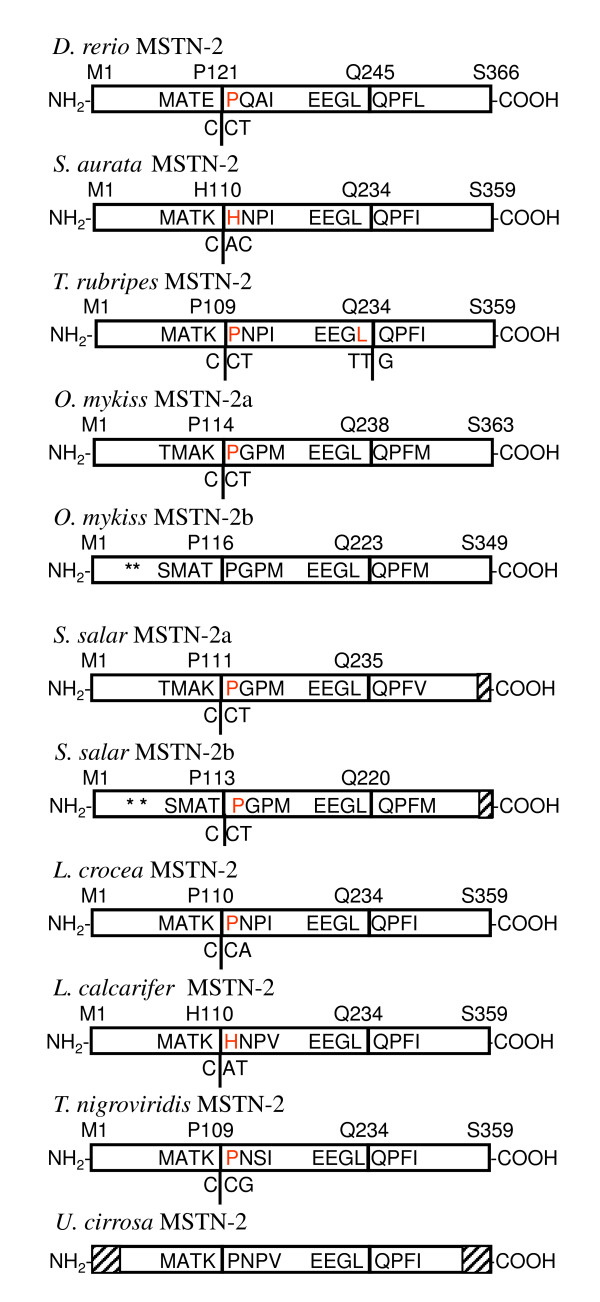
**Comparative mapping of exon boundaries in different fish *MSTN-2 *genes ***MSTN-2 *genes are organized into three exons. The three adjoining boxes for each protein represent the coding region for each exon. Amino acid sequences coded by exon boundaries are shown inside the boxes. The first amino acid coded by each exon is shown above, as is the last residue of the third exons. In all fish genes, the codon located at the first exonic boundary is partially coded by the first and second exons as shown (residue in red). When the exonic boundary is not known (genomic DNA sequence unknown) the residue is in black. Missing sequences due to partial cds appear in dashed boxes. Asterisks designate stop codon or deletion resulting in non-functional gene. Accession numbers: zebrafish (*Danio rerio*) *MSTN-2*, AY687474 (mRNA), DQ451548 (gene); gilthead sea bream (*Sparus aurata*) *MSTN-2*, AY046314 (mRNA), GQ379805 (gene, present study); fugu (*Takifugu rubripes*) *MSTN-2*, AY445321 (mRNA) ENSTRUG00000000132 (gene); rainbow trout (*Oncorhynchus mykiss*) *MSTN-2a*, DQ417326 (mRNA), DQ138301 (gene); rainbow trout (*Oncorhynchus mykiss*) *MSTN-2b*, DQ417327 (mRNA); Atlantic salmon (*Salmo salar *) *MSTN-2a*, EF392863 (gene, partial); Atlantic salmon (*Salmo salar *) *MSTN-2b*, EF392864 (gene, partial); large yellow croaker (*Pseudosciaena crocea *) *MSTN-2*, EU571244 (gene); barramundi (*Lates calcarifer*) *MSTN-2*, GU590863 (gene); spotted green pufferfish (*Tetraodon nigroviridis*), *MSTN-2*, ENSTNIT00000020527 (mRNA), ENSTNIG00000017160 (gene); shi drum (*Umbrina cirrosa*) *MSTN-2*, AY059386 (mRNA, partial).

Nevertheless, comparative analysis of *MSTN-2 *gene organization revealed a conserved organization among all nine fish species for which *MSTN-2 *was identified, including conserved pre-mRNA splice sites. The amino acids in the exon/intron junction of the second splice site are identical among the fishes studied and similar in their first splice site (Figure 8). The codon for proline or histidine at the first splice site is divided between exon 1 and exon 2 in all fishes.

## Conclusion

The gene and 5' flanking region of sa*MSTN-2 *gene were cloned and characterized. Evidence was provided of the polymorphism of both the promoter and the first intron among individuals of various populations. The two polymorphic loci are inherited in a Mendelian way and can be used to confirm the life history of fish in commercial farms. The results also suggest that this region underwent chromosomal rearrangements like duplication, insertions or deletions during the course of evolution.

## Authors' contributions

EB and BF conceived and initiated the project. EB carried out the cloning, sequence and genetic polymorphism analyses. Both authors read and approved the final manuscript.

## Supplementary Material

Additional file 1 **List of primers. **Names and sequences of primers used for sa*MSTN-2 *promoter and gene cloning, for sequencing and for polymorphism analysis. Click here for file

Additional file 2 **List of SNPs in sa*MSTN-2 *promoter alleles**. List of SNPs and small differences observed between alleles 'a', 'a_s_', 'b' and 'c' of sa*MSTN-2 *promoter, in the region extending from the translation start codon ATG until 1050 bp 5' upstream. Click here for file

Additional file 3 **List of SNPs in sa*MSTN-2 *gene. **List of SNPs and small differences observed in the exons and introns (1R, 2R and 4R) of sa*MSTN-2 *gene derived from sequences of several DNA samples. Click here for file

Additional file 4 **Polymorphism of sa*MSTN-2 *promoter in five DNA collections. **Genotype and allele frequencies of sa*MSTN-2 *promoter in five DNA collections. Click here for file

Additional file 5 **Polymorphism of sa*MSTN-2 *first intron in five DNA collections. **Genotype and allele frequencies of sa*MSTN-2 *intron-1 in five DNA collections. Click here for file
