# Supplementary material for: Myostatin-2 gene structure and polymorphism of the promoter and first intron in the marine fish Sparus aurata: evidence for DNA duplications and/or translocations
Source: BMC Genet. 2011 Feb 1;12:22. doi: 10.1186/1471-2156-12-22 (PMC3045353; doi:10.1186/1471-2156-12-22)
Supplement: Additional file 1 — List of primers. Names and sequences of primers used for saMSTN-2 promoter and gene cloning, for sequencing and for polymorphism analysis. [file 1471-2156-12-22-S1.DOC]

**Additional file 1. Names and sequences of primers used for sa*MSTN-2* promoter and gene**

**cloning, for sequencing and for polymorphism analysis**

| **Assigned Name** | Sequence | Purpose |
| --- | --- | --- |
| L1 | 5’-GTTCATCTTTACAAGCTAGCG-3’ | Cloning of sa*MSTN-2* promoter |
| L2 | 5’-TCCTGAACAATGCTGTGG-3’ | Cloning of sa*MSTN-*2 promoter |
| MSTNb-1 (rev) | 5’-GCTTGGAGGTCTGGTTCATC-3’ | Cloning of sa*MSTN-2* promoter |
| MSTNb-2 (rev) | 5’-CGAGCATCCTGGAGAGAA-3’ | Cloning of sa*MSTN-2* promoter |
| MSTNb-3 (rev) | 5’-TCCAACAGTAGCTCTGGGAAG-3’ | Sequencing of sa*MSTN-2* promoter |
| MSTNb-10 (fw) | 5’- ACCATGGCCTTGAATAGCAC-3’ | sa*MSTN-2* promoter allele ‘**b**’ specific |
| MSTNb-11 (fw) | 5’- GGCAGTTGTTTATCCGTCGT-3’ | sa*MSTN-2* promoter allele ‘**a**’ specific |
| MSTNb-13 (fw) | 5’- CGGCAAGGGTTATACGAAAA-3’ | sa*MSTN-2* promoter allele ‘**c**’ specific |
| MSTNb-14 (fw) | 5’- TGACGTCACCACAGAGGATT-3’ | sa*MSTN-2* promoter allele ‘**c**’ specific |
| MSTNb-2fw | 5’-TCTCTCCAGGATGCTCGTCT-3’ | Cloning of sa*MSTN-2* gene |
| MSTNb-7 (rev) | 5’-CTCTGTGATGGTCGGGTTTC-3’ | Cloning of sa*MSTN-2* gene |
| MSTN2-exon1-248 fw | 5’-AGCTCCTGGACCAGTACGAC-3’ | Sequencing of sa*MSTN-2* gene; Intron-1 polymorphism (EPIC-PCR) |
| MSTN2-intron1-501 fw | 5’-ATGCCATCTTTTGCACACTG-3’ | Sequencing of sa*MSTN-2* gene |
| MSTN2-exon2-56 fw | 5’-GAAGATCCAGCCCAAAAACA-3’ | Sequencing of sa*MSTN-2* gene |
| MSTN2-exon2-187 rev | 5’-CTGATTCGGGTGTTGTTTCC-3’ | Sequencing of sa*MSTN-2* gene;  5’RACE; Intron-1 polymorphism (EPIC-PCR) |
| MSTN2-exon2-261rev | 5’-GCAGCAGAGACTTGATGTCG-3’ | 5’RACE |
