# Supplementary material for: Myostatin-2 gene structure and polymorphism of the promoter and first intron in the marine fish Sparus aurata: evidence for DNA duplications and/or translocations
Source: BMC Genet. 2011 Feb 1;12:22. doi: 10.1186/1471-2156-12-22 (PMC3045353; doi:10.1186/1471-2156-12-22)
Supplement: Additional file 2 — List of SNPs in saMSTN-2 promoter alleles. List of SNPs and small differences observed between alleles 'a', 'as', 'b' and 'c' of saMSTN-2 promoter, in the region extending from the translation start codon ATG until 1050 bp 5' upstream. [file 1471-2156-12-22-S2.DOC]

**Additional file 2**. **List of SNPs and small differences observed between alleles ‘a’, ‘a_s_’, ‘b’ and ‘c’ of sa*MSTN-2* promoter, in the region extending from the translation start codon ATG until 1050 bp 5’ upstream**

Position number refers to allele ‘a’ sequence; position -1 corresponds to the first nucleotide preceding the translation start codon ATG; deletions are indicated by (-). nd, not determined.

| **Position** | **allele ‘a’** | **allele ‘a_s_’** | **allele ‘b’** | **allele ‘c’** |
| --- | --- | --- | --- | --- |
| -1049 | T | T | A | T |
| -1039 | T | T | T | C |
| -1035 | A | A | C | G |
| -1000 | C | A | A | A |
| -975 | C | A | A | A |
| -960 | A | A | A | - |
| -918 | A | T | T | T |
| -890 to -888 | GAC | --- | GAC | GAC |
| -886 | C | - | C | C |
| -868 | G | G | G | A |
| -866 | A | A | A | C |
| -832 -831 | CA | CA | GG | GG |
| -824 | A | A | - | A |
| -768 | G | G | A | A |
| -747 | A | A | G | A |
| -739 | T | T | G | T |
| -735 | T | A | T | A |
| -730 | G | G | A | G |
| -725 | A | T | T | T |
| -717 | A | G | G | G |
| -697 | G | G | A | G |
| -684 to -679 | TTTCTC | TT--- | ------ | TTTCTC |
| -677 to -673 | TTTTT | TTTTT | ----- | TTTTT |
| -666 | G | G | G | T |
| -652 | G | G | A | G |
| -633 | T | T | C | T |
| -614 | A | A | A | G |
| -559 | A | nd | C | A |
| -537 | G | nd | A | A |
| -439 | G | nd | G | A |
| -424 | C | nd | C | T |
| -397 | A | nd | A | C |
| -394 to -393 | GT | nd | GT | TG |
| -374 | G | nd | G | C |
| -316 | A | nd | A | G |
| -295 | T | nd | T | G |
| -293 | G | nd | G | A |
| -225 | C | nd | C | A |
| -222 | T | nd | T | TT |
| -216 | T | nd | T | C |
| -211 | T | nd | TT | TTAA |
| -156 | A | nd | A | T |
| -154 | C | nd | C | A |
| -117 | T | nd | T | A |
| -108 | A | nd | A | G |
| -97 | A | nd | A | G |
| -18 | C | nd | C | T |
