# Supplementary material for: Myostatin-2 gene structure and polymorphism of the promoter and first intron in the marine fish Sparus aurata: evidence for DNA duplications and/or translocations
Source: BMC Genet. 2011 Feb 1;12:22. doi: 10.1186/1471-2156-12-22 (PMC3045353; doi:10.1186/1471-2156-12-22)
Supplement: Additional file 3 — List of SNPs in saMSTN-2 gene. List of SNPs and small differences observed in the exons and introns (1R, 2R and 4R) of saMSTN-2 gene derived from sequences of several DNA samples. [file 1471-2156-12-22-S3.DOC]

**Additional file 3. List of SNPs and small differences observed in the exons and introns (1R, 2R and 4R) of sa*MSTN-2* gene derived from sequences of several DNA samples**

| **Nucleotide Position** | | **AY046314** | **2R- 17G** | **2R- 33** | **4R- 13RD** | **4R- 56,57RD** | **1R- 17G** | **1R- 3RD** | **Changes in**  **a.a.** |
| --- | --- | --- | --- | --- | --- | --- | --- | --- | --- |
| EXON1 | 44 | C | C | **T** | C | nd | **T** | **T** | S to F |
|  | 69 | C | C | T | C | nd | C | C | no |
|  | 216 | **A** | **A** | G | G | nd | G | G | no |
| INTRON 1 | intron1-16 | / | a | c | c | c | c | c | / |
|  | intron1-30 | / | g | g | g | a | - | - | / |
|  | intron1-31 | / | a | t | a | t | - | - | / |
|  | intron1-72 | / | a | a | g | g | - | - | / |
|  | intron1-75 | / | c | - | c | c | - | - | / |
|  | intron1-88 | / | g | c | c | c | - | - | / |
|  | intron1-131 | / | c | c | a | a | - | - | / |
|  | intron1-225 | / | t | t | c | c | t | t | / |
|  | intron1-265 | / | a | a | - | - | a | a | / |
|  | intron1-309-310 | / | cc | cc | tg | tg | cc | cc | / |
|  | intron1-313 | / | c | c | a | a | c | c | / |
|  | intron1-325 | / | c | c | tt | tt | c | c | / |
|  | intron1-329 | / | t | t | a | a | t | t | / |
|  | intron1-332 | / | c | c | g | g | c | c | / |
|  | intron1-346 | / | g | g | t | t | g | g | / |
|  | intron1-363 | / | c | c | g | g | c | c | / |
|  | intron1-388 | / | c | c | t | t | c | c | / |
|  | intron1-427 | / | g | g | a | a | g | g | / |
|  | intron1-437 | / | c | c | g | g | c | c | / |
|  | intron1-440 | / | t | t | a | a | t | t | / |
|  | intron1-459 | / | t | t | a | a | t | t | / |
|  | intron1-482 | / | a | a | c | c | a | a | / |
|  | intron1-523 | / | t | t | c | c | t | t | / |
|  | intron1-530 | / | t | t | g | g | t | t | / |
|  | intron1-552 | / | g | g | c | c | g | g | / |
|  | intron1-562 | / | g | g | a | a | g | g | / |
|  | intron1-586 | / | c | c | a | a | c | c | / |
|  | intron1-614 | / | g | g | c | c | g | g | / |
|  | intron1-618-620 | / | cat | cat | --- | --- | cat | cat | / |
|  | intron1-670 | / | c | c | g | g | c | c | / |
|  | intron1-736 | / | a | a | g | g | a | a | / |
|  | intron1-742 | / | t | t | c | c | t | t | / |
|  | intron1-755 | / | a | a | g | g | a | a | / |
|  | intron1-766 | / | g | g | c | c | g | g | / |
|  | intron1-812 | / | t | t | c | c | t | t | / |
|  | intron1-817 | / | c | c | t | t | c | c | / |
|  | intron1-822 | / | t | t | c | c | t | t | / |
|  | intron1-839 | / | g | g | a | a | g | g | / |
|  | intron1-857 | / | c | c | g | g | c | c | / |
|  | intron1-879 | / | t | t | a | a | t | t | / |
|  | intron1-892 | / | c | c | g | g | c | c | / |
|  | intron1-894 | / | t | t | c | c | t | t | / |
|  | intron1-910 | / | t | t | a | a | t | t | / |
|  | intron1-930 | / | t | t | ttt | ttt | t | t | / |

**Additional file 3 (continued)**

| **Nucleotide Position** | | **AY046314** | **2R- 17G** | **2R- 33** | **4R- 13RD** | **4R- 56,57RD** | **1R- 17G** | **1R- 3RD** | **Changes in**  **a.a.** |
| --- | --- | --- | --- | --- | --- | --- | --- | --- | --- |
| EXON 2 | 333 | T | T | T | **C** | **C** | T | T | no |
|  | 411 | C | C | C | T | T | C | C | no |
|  | 420 | G | G | G | T | G | G | G | no |
|  | 433 | C | C | C | A | A | C | C | no |
|  | 442 | G | G | G | A | A | G | G | no |
|  | 456 | C | C | C | T | T | C | C | no |
|  | 489 | G | **A** | **A** | **A** | **A** | **A** | **A** | no |
|  | 522 | T | T | T | **G** | nd | T | T | no |
|  | 552 | T | T | T | **C** | nd | T | T | no |
|  | 564 | A | A | A | G | nd | A | A | no |
|  | 567 | T | T | T | C | nd | T | T | no |
|  | 570 | C | C | C | T | nd | C | C | no |
|  | 684 | G | G | G | A | nd | G | G | no |
| INTRON 2 | intron2-3 | / | a | a | g | nd | a | a | / |
|  | intron2-7 | / | g | g | c | nd | g | g | / |
|  | intron2-23 | / | c | c | t | nd | c | c | / |
|  | intron2-43 | / | c | c | - | nd | - | c | / |
|  | intron2-63 | / | c | c | t | nd | c | c | / |
|  | intron2- 72-74 | / | ggt | ggt | tac | nd | ggt | ggt | / |
|  | intron2-112 | / | t | t | a | nd | t | t | / |
|  | intron2-170 | / | g | g | a | nd | g | g | / |
|  | intron2-185 | / | a | a | t | nd | a | a | / |
|  | intron2-187 | / | a | a | c | nd | a | a | / |
|  | intron2-189 | / | t | t | tgcgt | nd | t | t | / |
|  | intron2-246 | / | c | c | t | nd | c | c | / |
|  | intron2-250 | / | g | g | a | nd | g | g | / |
|  | intron2-255 | / | g | g | a | nd | g | g | / |
|  | intron2-313 | / | g | g | t | nd | g | g | / |
|  | intron2-335 | / | c | c | t | nd | c | c | / |
| EXON 3 | 723 | C | C | C | A | nd | C | C | no |
|  | 774 | A | **G** | **G** | **G** | nd | **G** | **G** | no |
|  | 813 | C | C | C | **T** | nd | C | C | no |
|  | 831 | T | T | T | **C** | nd | T | T | no |
|  | 861 | G | G | G | **C** | nd | G | G | no |
|  | 870 | C | C | A | C | nd | C | C | no |
|  | 882 | A | A | A | **G** | nd | A | A | no |
|  | 933 | G | G | G | **A** | nd | G | G | no |
|  | 939 | A | A | A | **G** | nd | A | A | no |
|  | 975 | G | G | G | **A** | nd | G | G | no |
|  | 978 | C | C | C | **A** | nd | C | C | no |
|  | 1029 | T | T | T | **C** | nd | T | T | no |
|  | 1035 | C | C | C | **T** | nd | C | C | no |

Positions in the introns are according to 2R-17G sequence; positions in the exons are according to the published cDNA sequence Accession number AY046314.

Most of the SNPs are based on more than one PCR reaction or cloned DNA.

nd, not determined; in bold, SNPs found also in a cDNA from another individual.
