# Supplementary material for: Myostatin-2 gene structure and polymorphism of the promoter and first intron in the marine fish Sparus aurata: evidence for DNA duplications and/or translocations
Source: BMC Genet. 2011 Feb 1;12:22. doi: 10.1186/1471-2156-12-22 (PMC3045353; doi:10.1186/1471-2156-12-22)
Supplement: Additional file 4 — Polymorphism of saMSTN-2 promoter in five DNA collections. Genotype and allele frequencies of saMSTN-2 promoter in five DNA collections. [file 1471-2156-12-22-S4.DOC]

| DNA collection | Fish size | N | Genotype frequency | | | | | | | | | | Allele frequency | | | |
| --- | --- | --- | --- | --- | --- | --- | --- | --- | --- | --- | --- | --- | --- | --- | --- | --- |
|  |  |  | Homozygotes | | | | Heterozygotes | | | | | |  |  |  |  |
|  |  |  | a/a | b/b | c/c | **Total** | a/b | b/c | a/c | as/b | as/c | **Total** | a | b | c | as |
| Ardag Fisheries  (Israel) | Large | 30 | 0  (0%) | 17  (56.7%) | 2  (6.7%) | 19  (63.3%) | 1  (3.3%) | 10  (33.3%) | 0  (0%) | 0  (0%) | 0  (0%) | 11  (36.7%) | 1  (1.7%) | 45  (75.0%) | 14  (23.3%) | 0  (0%) |
|  | Small | 30 | 0  (0%) | 17  (56.7%) | 2  (6.7%) | 19  (63.3%) | 0  (0%) | 11  (36.7%) | 0  (0%) | 0  (0%) | 0  (0%) | 11  (36.7%) | 0  (0%) | 45  (5.0%) | 15  (25.0%) | 0  (0%) |
|  | **Total** | 60 | 0  (0%) | 34  (56.7%) | 4  (6.7%) | 38  (63.3%) | 1  (1.7%) | 21  (35.0%) | 0  (0%) | 0  (0%) | 0  (0%) | 22  (36.7%) | 1  (0.8%) | 90  (75.0%) | 29  (24.2%) | 0  (0%) |
| Atlit Fisheries  (Israel) | Large | 27 | 0  (0%) | 10  (37.0%) | 2  (7.4%) | 12  (44.4%) | 2  (7.4%) | 10  (37.0%) | 2  (7.4%) | 1  (3.7%) | 0  (0%) | 15  (55.6%) | 4  (7.4%) | 33  (61.1%) | 16  (29.6%) | 1  (1.9%) |
|  | Average | 25 | 0  (0%) | 9  (36.0%) | 0  (0%) | 9  (36.0%) | 5  (20.0%) | 7  (28.0%) | 2  (8.0%) | 1  (4.0%) | 1  (4.0%) | 16  (64.0%) | 7  (14.0%) | 31  (62.0%) | 10  (20.0%) | 2  (4.0%) |
|  | **Total** | 52 | 0  (0%) | 19  (36.5%) | 2  (3.8%) | 21  (40.4%) | 7  (13.5%) | 17  (32.7%) | 4  (7.7%) | 2  (3.8%) | 1  (1.9%) | 31  (59.6%) | 11  (10.6%) | 64  (61.5%) | 26  (25.0%) | 3  (2.9%) |
| Faro Fisheries  (Portugal) | All | 19 | 0  (0%) | 7  (36.8%) | 0  (0%) | 7  (36.8%) | 0  (0%) | 12  (63.2%) | 0  (0%) | 0  (0%) | 0  (0%) | 12  (63.2%) | 0  (0%) | 26  (68.4%) | 12  (31.6%) | 0  (0%) |
| Wild samples  (Italy) | All | 23 | 0  (0%) | 10  (43.5%) | 0  (0%) | 10  (43.5%) | 4  (17.4%) | 8  (34.8%) | 0  (0%) | 1  (4.3%) | 0  (0%) | 13  (56.5%) | 4  (8.7%) | 33  (71.7%) | 8  (17.4%) | 1  (2.2%) |
| "GC" samples  (Israel) | All | 15 | 0  (0%) | 7  (46.7%) | 0  (0%) | 7  (46.7%) | 2  (13.3%) | 4  (26.7%) | 0  (0%) | 2  (13.3%) | 0  (0%) | 8  (53.3%) | 2  (6.7%) | 22  (73.3%) | 4  (13.3%) | 2  (6.7%) |
| **TOTAL** |  | 169 | 0  (0%) | 77  (45.6%) | 6  (3.6%) | 83  (49.1%) | 14  (8.3%) | 62  (36.7%) | 4  (2.4%) | 5  (3.0%) | 1  (0.6%) | 86  (50.9%) | 18  (5.3%) | 235  (69.5%) | 79  (23.4%) | 6  (1.8%) |

**Additional file 4. Analysis of sa*MSTN-2* promoter polymorphism in five DNA collections**

Alleles of sa*MSTN-2* promoter were determined by PCR amplification with the allele-specific primer sets MSTNb-10/MSTNb-1, MSTNb-11/MSTNb-1 and MSTNb-13/MSTNb-2 (specific to allele ‘b’, ‘a’, and ‘c’, respectively). Allele ‘as’ is a shorter PCR fragment obtained with the ‘a’-specific primers. Five genomic *S. aurata* DNA collections were analyzed. In parentheses, percent frequencies.
