# Supplementary material for: Myostatin-2 gene structure and polymorphism of the promoter and first intron in the marine fish Sparus aurata: evidence for DNA duplications and/or translocations
Source: BMC Genet. 2011 Feb 1;12:22. doi: 10.1186/1471-2156-12-22 (PMC3045353; doi:10.1186/1471-2156-12-22)
Supplement: Additional file 5 — Polymorphism of saMSTN-2 first intron in five DNA collections. Genotype and allele frequencies of saMSTN-2 intron-1 in five DNA collections. [file 1471-2156-12-22-S5.DOC]

**Additional file 5. Analysis of sa*MSTN-2* intron-1 polymorphism in five DNA collections**

| DNA collection | Fish size | N | Genotypes Frequency | | | | | | | | Allele frequency | | |
| --- | --- | --- | --- | --- | --- | --- | --- | --- | --- | --- | --- | --- | --- |
|  |  |  | Homozygotes | | | | Heterozygotes | | | |  |  |  |
|  |  |  | 1R/1R | 2R/2R | 4R/4R | **Total** | 1R/2R | 2R/4R | 1R/4R | **Total** | 1R | 2R | 4R |
| Ardag Fisheries  (Israel) | Large | 30 | 0  (0%) | 8  (26.7%) | 7  (23.3%) | 15  (50.0%) | 1  (3.3%) | 14  (46.7%) | 0  (0%) | 15  (50.0%) | 1  (1.7%) | 31  (51.7%) | 28  (46.7%) |
|  | Small | 30 | 0  (0%) | 10  (33.3%) | 10  (33.3%) | 20  (66.7%) | 0  (0%) | 10  (33.3%) | 0  (0%) | 10  (33.3%) | 0  (0%) | 30  (50.0%) | 30  (50.0%) |
|  | **Total** | 60 | 0  (0%) | 18  (30.0%) | 17  (28.3%) | 35  (58.3%) | 1  (1.7%) | 24  (40.0%) | 0  (0%) | 25  (41.7%) | 1  (0.8%) | 61  (50.8%) | 58  (48.3%) |
| Atlit Fisheries  (Israel) | Large | 27 | 0  (0%) | 16  (59.3%) | 1  (3.7%) | 17  (63.0%) | 2  (7.4%) | 7  (25.9%) | 1  (3.7%) | 10  (37.0%) | 3  (5.6%) | 41  (75.9%) | 10  (18.5%) |
|  | Average | 25 | 0  (0%) | 14  (56.0%) | 0  (0%) | 14  (56.0%) | 2  (8.0%) | 9  (36.0%) | 0  (0%) | 11  (44.0%) | 2  (4.0%) | 39  (78.0%) | 9  (18.0%) |
|  | **Total** | 52 | 0  (0%) | 30  (57.7%) | 1  (1.9%) | 31  (59.6%) | 4  (7.7%) | 16  (30.8%) | 1  (1.9%) | 21  (40.4%) | 5  (4.8%) | 80  (76.9%) | 19  (18.3%) |
| Faro Fisheries  (Portugal) | All | 19 | 0  (0%) | 13  (68.4%) | 0  (0%) | 13  (68.4%) | 0  (0%) | 6  (31.6%) | 0  (0%) | 6  (31.6%) | 0  (0%) | 32  (84.2%) | 6  (15.8%) |
| Wild samples  (Italy) | All | 23 | 0  (0%) | 12  (52.2%) | 0  (0%) | 12  (52.2%) | 3  (13.0%) | 8  (34.8%) | 0  (0%) | 11  (47.8%) | 3  (6.5%) | 35  (76.1%) | 8  (17.4%) |
| "GC" samples  (Israel) | All | 15 | 0  (0%) | 7  (46.7%) | 0  (0%) | 7  (46.7%) | 2  (13.3%) | 6  (40.0%) | 0  (0%) | 8  (53.3%) | 2  (6.7%) | 22  (73.3%) | 6  (20.0%) |
| **TOTAL** |  | 169 | 0  (0%) | 80  (47.3%) | 18  (10.6%) | 98  (58.0%) | 10  (5.9%) | 60  (35.5%) | 1  (0.6%) | 71  (42.0%) | 11  (3.2%) | 230  (68.0%) | 97  (28.7%) |

Alleles of sa*MSTN-2* intron-1 were determined by EPIC-PCR amplification using primers MSTN-2-exon1-248fw and MSTN-2-exon2-187rev, corresponding to intron-1 flanking exons. In parentheses, percent frequencies.
